# Pooled genetic screens with image‐based profiling

**DOI:** 10.15252/msb.202110768

**Published:** 2022-11-11

**Authors:** Russell T Walton, Avtar Singh, Paul C Blainey

**Affiliations:** ^1^ Broad Institute of MIT and Harvard Cambridge MA USA; ^2^ Department of Biological Engineering MIT Cambridge MA USA; ^3^ Koch Institute for Integrative Cancer Research MIT Cambridge MA USA; ^4^ Present address: Department of Cellular and Tissue Genomics Genentech South San Francisco CA USA

**Keywords:** morphological profiling, multiplexed imaging, optical microscopy profiling, optical screening, pooled genetic screening, Biotechnology & Synthetic Biology, Methods & Resources

## Abstract

Spatial structure in biology, spanning molecular, organellular, cellular, tissue, and organismal scales, is encoded through a combination of genetic and epigenetic factors in individual cells. Microscopy remains the most direct approach to exploring the intricate spatial complexity defining biological systems and the structured dynamic responses of these systems to perturbations. Genetic screens with deep single‐cell profiling via image features or gene expression programs have the capacity to show how biological systems work in detail by cataloging many cellular phenotypes with one experimental assay. Microscopy‐based cellular profiling provides information complementary to next‐generation sequencing (NGS) profiling and has only recently become compatible with large‐scale genetic screens. Optical screening now offers the scale needed for systematic characterization and is poised for further scale‐up. We discuss how these methodologies, together with emerging technologies for genetic perturbation and microscopy‐based multiplexed molecular phenotyping, are powering new approaches to reveal genotype–phenotype relationships.

## Graphical abstract

This review discusses the technological advances that enable studies of genotype‐to‐phenotype relationships with microscopy‐based imaging and proposes a roadmap for the future development and application of pooled profiling screens with image‐based phenotypes.

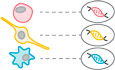

## Introduction

**Figure 1:**
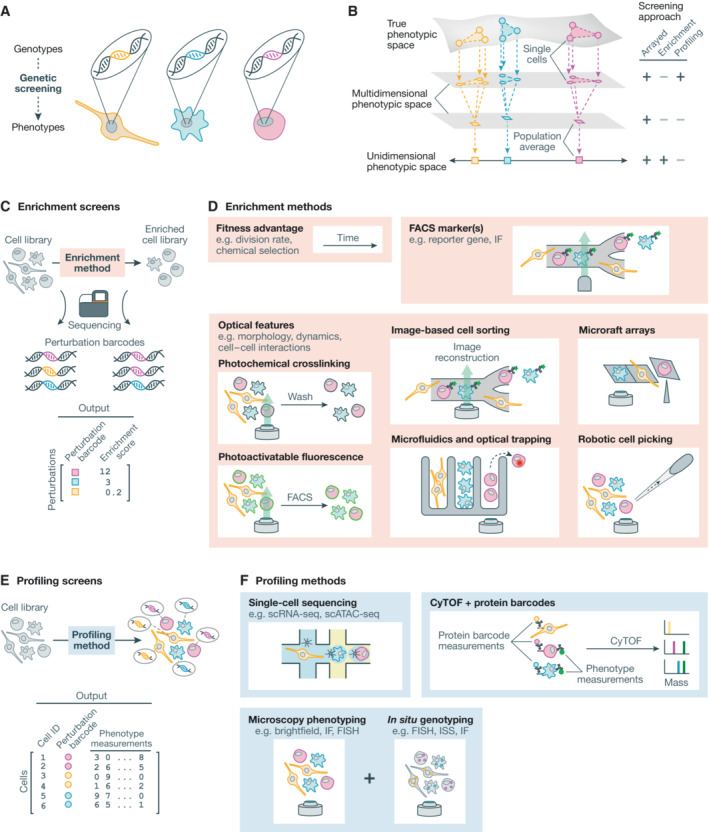
Approaches to genetic screening (A) Genetic screens seek to map genotypes to the phenotypes they produce. (B) Screening methodologies capture projections of cell phenotypes. Pooled profiling screens project individual cells into a multidimensional phenotypic space defined by the profiling method. Pooled enrichment screens project population averages into a unidimensional phenotypic space defined by the enrichment criteria. Arrayed screens can embody either of these phenotype–genotype associations. (C) Enrichment screens subject an initial cell library to an enrichment process to select for a phenotype of interest. Perturbation enrichment is determined by comparing the abundance of perturbation barcodes in the cell library before and after selection using next generation sequencing. (D) Cells can be enriched through a fitness advantage, fluorescence‐activated cell sorting, or one of several approaches to isolate cells based on microscopy‐defined features. (E) Profiling screens subject a complete cell library to profiling. Individual cells are assigned both perturbations and multidimensional phenotypic measurements. (F) Single cell profiling methods for genetic screening include single‐cell sequencing approaches, CyTOF using protein barcodes, and microscopy‐based phenotyping with *in situ* genotyping. FACS, fluorescence‐activated cell sorting; IF, immunofluorescence; scRNA‐seq, single‐cell RNA sequencing; scATAC‐seq, single‐cell assay for transposase‐accessible chromatin using sequencing; CyTOF, cytometry by time‐of‐flight.

The genetics and epigenetics of interacting cells over developmental time give rise to organisms and their characteristics. Understanding how genotypes give rise to phenotypes is the core objective of forward genetic screening, a set of approaches that systematically perturb the genome and record the phenotypic consequences (Fig 1A; Doench, 2018; Schuster *et al*, 2019). Genetic screens have a broad set of applications, including uncovering fundamental biology, characterizing the function of sequence variants, and identifying the molecular targets of drug candidates. The measurement of spatiotemporally resolved visual phenotypes in genetic screens, sampling the vast and dynamic structural complexity of biological systems, provides an information‐rich basis to explore genotype–phenotype relationships.

Today, many screening approaches apply targeted genotypic perturbations across an otherwise constant genetic background, such that differential phenotypes can be directly attributed to the perturbation. For example, consider a CRISPR‐Cas9 gene knockout (CRISPR KO) screen in which a Cas9 nuclease is directed by a sequence‐programmable guide RNA (gRNA) to a complementary genomic target, generating mutations that ablate function of the target gene. Here, the genotype of a cell is defined by the gene that has been targeted for loss‐of‐function. Following perturbation, the objective is to understand how each genotype influences cell phenotype. The measured phenotype could take the form of a unidimensional measurement to capture a specific feature of interest, such as relative cell fitness in a population, or a high‐dimensional measurement to capture multiple aspects of cell phenotype, like a visual phenotype or transcriptional state (Fig 1B). In the example CRISPR KO screen, each gene loss‐of‐function could be connected to cellular abundance as a proxy for gene essentiality.

Genetic screening approaches fundamentally differ in the way perturbations and phenotypes are associated (Fig 1B). Strategies can be classified into three groups: arrayed, pooled enrichment, and pooled profiling screens. In arrayed screens, perturbations are identified by position in a multiwell plate and phenotypic measurements are made for each well. The logistics of working with hundreds to tens of thousands of individual samples pose a major challenge to many researchers' ability to implement large‐scale arrayed screens. Pooled screens offer a solution to this problem by introducing a large number of perturbations into a single sample. In pooled enrichment screens, cells of interest are then enriched (e.g., by survival) and next‐generation sequencing (NGS) is used to compare the abundance of “perturbation barcodes”—sequences that encode perturbation identity—before and after enrichment (Fig 1C and D). In CRISPR screens, the gRNA itself may conveniently function as a perturbation barcode. Finally, in pooled profiling screens, phenotypic features and perturbation barcodes are measured in each individual cell in the mixed population (Fig 1E).

While image‐based “visual” phenotypes have largely been inaccessible in pooled genetic screening formats, technological advances now provide options for assaying microscopy‐defined phenotypes in pooled screens. In this review, we discuss technological advances that enable studies of genotype‐to‐phenotype relationships with microscopy‐based imaging. We provide an overview of approaches for arrayed, pooled enrichment, and pooled profiling screens with visual phenotypes and focus on the current suite of perturbation technologies and microscopy‐based phenotyping approaches, in particular as they apply to pooled profiling screens. Finally, we suggest a roadmap for continued development and application of pooled profiling screens to extend the impact of microscopy‐based genetic screening.

## Microscopy‐based genetic screening maps genotypes to visual phenotypes

### Arrayed screens

Arrayed screens allow the greatest flexibility in choice of perturbation and phenotyping approaches thanks to the simplicity of perturbation association to cell sample by position in the arrayed layout, for example a multiwell plate (Fig 1B). This is an important contrast with pooled screens (discussed in the following section) where more complex designs and extra steps are necessary to deconvolute the pooled perturbations. While maintaining compatibility with barcoded perturbations that are required for pooled screens, arrayed screens can additionally employ RNA perturbants without DNA precursors, such as small interfering RNA (siRNA) or CRISPR ribonucleoproteins, and chemical perturbants (Chia *et al*, 2010; Serçin *et al*, 2019). Phenotypic measurements may be perturbation‐averaged, by taking a bulk measurement of all cells in a well, or single‐cell resolution, via microscopy or single‐cell sequencing approaches. Such measurements can span dimensionality from a single fluorescent reporter to molecular omics measurements.

The simplicity and flexibility of implementation make arrayed screens an attractive approach at relatively small scales. However, generation and maintenance of large arrayed cell libraries is challenging, expensive, and requires particular care to limit plate‐position and plate‐to‐plate statistical biases. Further, when control cells and perturbed cells are segregated in different wells, confounding epiphenomena may obscure perturbation‐specific effects. At large scales, arrayed screens require complex and costly automation, large teams, and extensive validation procedures; for smaller teams, pooled screens may be the only feasible option.

Despite the challenges, genome‐wide arrayed screens have produced valuable data through large‐scale efforts. For example, Boutros *et al* (2004) conducted a genome‐wide growth and viability screen in Drosophila cell lines, identifying hundreds of essential genes. And, in a genome‐wide arrayed siRNA screen in human embryonic stem cells using a fluorescent reporter of pluripotency, Chia *et al* (2010) identified genes responsible for the maintenance of pluripotency. More recently, genome‐wide arrayed CRISPR‐KO screens have been performed in primary kidney fibroblasts to identify relevant factors in kidney disease (Turner *et al*, 2020). Arrayed screens in their diverse forms have been reviewed in greater detail elsewhere (Zanella *et al*, 2010; Boutros *et al*, 2015).

### Pooled screens

The major advantages of pooled screens over arrayed formats are that cell libraries can be generated, maintained, and screened as single samples, and that perturbation effects are determined using robust within‐sample comparisons. Pooled oligo libraries encoding genetic perturbation reagents are commercially available at flexible scales from a range of vendors and enable a straightforward and cost‐effective path to realizing a specified cell library. In a typical workflow, these oligos can be cloned into lentiviral packaging vectors, prepared as a lentiviral library, and transduced into the screening cell line to generate the cell library, each step in a single pooled reaction. This cell library can then be maintained and screened as a single culture. In addition to the reduced experimental burden of pooled screens, the handling of fewer individual cultures and the presence of internal controls in the mixed cell populations help reduce batch variability, avoid confounds, and improve statistical power. Mixing differently perturbed cells throughout the same sample is a key advantage for profiling studies where the comparison of perturbations against one another is often of interest. Despite these advantages, achieving sufficient scale to provide reliable estimates of genotype–phenotype associations yet challenges many pooled screening efforts. A typical CRISPR KO screen of 20,000 genes (roughly every single‐gene knockout in the human genome) with four gRNAs per gene with an average coverage of 200 cells per gRNA requires obtaining data from 16 million cells. The way that pooled screens extract perturbation‐phenotype associations from a mixed population is the critical distinguishing factor between the two categories of pooled screens: enrichment screens and profiling screens.

#### Pooled enrichment screens

Pooled enrichment screens employ selection for a pre‐defined phenotype of interest to yield a scalar enrichment score for each perturbation in a library. Enrichment scores are determined by NGS of perturbation barcodes to compare their abundance in the starting and enriched cell libraries (Fig 1B and C). As the phenotype of interest determines the experimental enrichment strategy, it is necessary to strictly define the phenotype prior to screening. While many individual cells receive each perturbation, the enrichment score reflects an average of the enrichment across individual cells. Thus, enrichment scores describe population‐averaged phenotypes, in contrast to phenotypes measured in individually genotyped cells.

Enrichment screens can select for complex phenotypes, including complex multiparametric image‐based phenotypes; however, they necessarily project these phenotypes into unidimensional space represented by the enrichment score. For example, viability screens typically compress several phenotypes including cell division rate, cell–cell signaling, tolerance of various cellular stresses, and even adherence to cultureware, to a single “fitness score” determined by the endpoint NGS guide abundance measurement. Genome‐scale pooled enrichment screens have become routine using straightforward enrichment methods including those targeting fitness/viability effects and fluorescence‐activated cell sorting (FACS) on the scalar signals of reporter genes or antibody stains. For example, the Cancer Dependency Map project encompasses genome‐wide pooled fitness enrichment screens performed in 501 and 908 cancer cell lines with RNAi and CRISPR KO perturbations, respectively (Tsherniak *et al*, 2017; Pacini *et al*, 2021).

There exists a growing set of methods to enable the enrichment of cells on more complex functional, molecular, and morphological axes (Fig 1D). In addition to the now‐conventional fitness advantage and FACS methodologies, recent advances in microscopy‐based approaches have extended enrichment screens to complex optical phenotypes including subcellular localization of biomolecules and cell morphology. And several such approaches maintain live cells following enrichment, an additional attractive feature enabling further characterization of the population of interest.

One set of approaches leverages photochemical reactions to selectively label individual cells as they are imaged with fluorescence microscopy. This labeling can enable enrichment of cells based on a microscopy‐defined phenotype. The Photostick method relies on a photochemical crosslinker that enables selected cells to remain adhered while unselected cells are enzymatically removed (Chien *et al*, 2015). Several groups have also developed approaches using selective photoconversion of fluorescent proteins based on visual phenotypes measured with microscopy for subsequent enrichment with FACS (Kuo *et al*, 2016; Hasle *et al*, 2020; Kanfer *et al*, 2021; Yan *et al*, 2021). Hasle *et al* (2020) employed a photoconversion method termed visual cell sorting to screen 346 SV40 nuclear localization sequence variants across about 638,000 cells, identifying variants with improved nuclear localization relative to the wild‐type sequence. Kanfer *et al* (2021) used a photoactivatable fluorescent protein to label cells for FACS isolation with TFEB localization phenotypes in a genome wide screen, imaging over 12 million cells. Yan *et al* (2021) screened over 11 million cells in a library of about 6,000 perturbations, isolating cells displaying nuclear size phenotypes through a photoactivatable fluorescent protein and subsequent cell sorting.

In addition to microscopy‐based approaches, specialized cell sorters have been developed to reconstruct fluorescence or Raman microscopy images of cells and sort cells on image‐based criteria in real time (Nitta *et al*, 2018, 2020; preprint: Salek *et al*, 2022; Schraivogel *et al*, 2022). These approaches have been demonstrated with diverse phenotypic measurements, including fluorescent reporter localization and surface epitope immunofluorescence (IF) in live cells and intracellular IF in fixed cells. Schraivogel *et al* (2022) leveraged fluorescence image‐based cell sorting to perform a genome‐wide screen in HeLa cells to identify factors regulating the localization of p65, a key component of the nuclear factor κB pathway. At the demonstrated flow rate, this approach would enable genome‐wide screens with 3 gRNAs per gene and 100× coverage per gRNA in just 9 h of sorting time.

Robotic cell picking, microraft arrays, and optical trapping in microfluidic chips have also been used to mechanically isolate cells based on optical phenotypes (Piatkevich *et al*, 2018; Luro *et al*, 2020; Wheeler *et al*, 2020). Piatkevich *et al* (2018) developed a robotic cell picking approach to select cells based on visual phenotypes and employed the system to perform directed evolution of fluorescent proteins. Wheeler *et al* (2020) used automated confocal microscopy and microraft arrays to screen over 12,000 perturbations in about 120,000 cells, identifying RNA binding proteins involved in stress granule formation. Luro *et al* (2020) used a microfluidic chip to screen genetic circuits in *Escherichia coli*, making live‐cell measurements of circuit activity with microscopy and using optical trapping to isolate selected cells for genotyping.

While enrichment screens represent phenotype with an enrichment score, a unidimensional and population‐averaged metric, some experimental designs for enrichment screening can expand these capabilities. Subjecting the same cell library to multiple distinct enrichment and readout steps will yield multiple enrichment scores (Surdziel *et al*, 2017). However, obtaining each distinct enrichment score set essentially requires performing a complete additional screen. An exception could include the use of picking or photoconversion methods with multiple sorting bins, though the number of bins and/or capacity of the sorter would place a limit on the number of simultaneous enrichments that could be performed (Hasle *et al*, 2020). Additionally, the resolution of perturbation‐phenotype association can be improved by pairing perturbation barcodes with randomized clonal barcodes. Clonal barcodes uniquely identify the original perturbed cells such that the enrichment score of each clone can be measured separately to segregate clonal effects and provide some distribution‐level information. Clonal barcodes have been implemented with CRISPR‐Cas9‐based perturbations through the addition of a randomized barcode to the gRNA (Schmierer *et al*, 2017; Zhu *et al*, 2019). Clonal barcoding offers a compromise between perturbation‐averaged and single‐cell resolution phenotypes, distinguishing among some sources of cell variability, such as genetic heterogeneity or semi‐random perturbation outcomes, but not others, like cell cycle stage, local spatial context, and other sources of biological noise.

#### Pooled profiling screens

**Figure 2:**
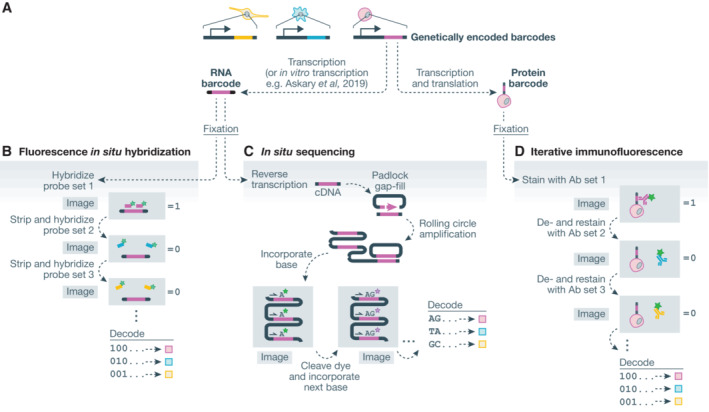
Approaches to measure perturbation barcodes *in situ* (A) Perturbation barcodes are genetically encoded and may be transcribed to RNA or transcribed and translated to protein epitopes. (B) Fluorescence *in situ* hybridization approaches measure RNA barcodes through iterative hybridization, imaging, and stripping of fluorescent probes. Diverse encoding schemes may be used. (C) *In situ* sequencing approaches clonally amplify barcode sequences *in situ* and read out barcodes through iterative cycles of sequencing/imaging. (D) Iterative immunofluorescence approaches detect protein barcodes—unique combinations of epitopes—by iteratively staining, imaging, and destaining with fluorescently labeled antibodies. Epitopes are used combinatorially in each barcode and diverse encoding schemes may be used.

Pooled profiling screens capture perturbation barcodes and high‐dimensional phenotypes of individual cells in a population (Fig 1B and E). The three broad approaches to pooled profiling screens employ single‐cell “omic” sequencing, mass spectrometry, and microscopy as their foundational technologies (Fig 1F). Single‐cell sequencing screens adapt single‐cell RNA sequencing (scRNA‐seq) or single‐cell assay for transposase‐accessible chromatin sequencing (scATAC‐seq) to recover perturbation barcodes alongside the phenotypic measurements (Adamson *et al*, 2016; Dixit *et al*, 2016; Jaitin *et al*, 2016; Datlinger *et al*, 2017; Rubin *et al*, 2019; Replogle *et al*, 2020). Mass spectrometry approaches require encoding perturbation barcodes at the protein level as unique epitope combinations are required for readout using methods including cytometry by time of flight (CyTOF) and multiplexed ion beam imaging by time of flight (MIBI‐TOF) (Keren *et al*, 2019) to characterize both genotype and phenotype at the protein level (Wroblewska *et al*, 2018; Dhainaut *et al*, 2022). Microscopy‐based pooled profiling screens use a variety of barcoding and imaging approaches to measure both cell phenotype and genotype *in situ* (Fig 2A–D; Emanuel *et al*, 2017; Lawson *et al*, 2017; Feldman *et al*, 2019; Wang *et al*, 2019; Shi *et al*, 2020; Dhainaut *et al*, 2022). Three general approaches for *in situ* genotyping have been demonstrated: fluorescence *in situ* hybridization (FISH), *in situ* sequencing (ISS), and iterative IF (Fig 2B–D).

In FISH genotyping approaches, perturbation barcodes are transcribed in live cells, a signal amplification step generating many barcode copies in each cell (Fig 2A). Cells are then fixed and barcodes are measured by iteratively hybridizing fluorescent probes, imaging cells, stripping probes, and repeating with subsequent probe sets until all barcodes can be decoded (Fig 2B). Alternatively, transcriptionally inactive barcodes have also been measured following *in vitro* transcription in fixed cells (Askary *et al*, 2019). The first microscopy‐based pooled screening methods were described in two 2017 *E. coli* screening studies that measured perturbation barcodes with FISH (Emanuel *et al*, 2017; Lawson *et al*, 2017). Lawson *et al* (2017) conducted a screen of three *E. coli* variants and measured complex phenotypes including over 4 h of dynamics by tracking bacteria in a microfluidic chip. Emanuel *et al* (2017) screened 60,000 fluorescent protein variants for brightness and stability while retrieving perturbation barcodes by multiplexed FISH. Though characterizing a simple phenotype, the screen profiled 20 million individual bacteria. And in a screen of lncRNA localization, Wang *et al* (2019) knocked out 54 genes encoding RNA‐binding proteins and profiled about 30,000 human osteosarcoma cells, characterizing both phenotype and genotype with multiplexed FISH. In an alternative approach for highly multiplexed barcode detection, Shi *et al* (2020) developed a hyperspectral imaging‐based FISH method, termed HiPR‐FISH, using 10 fluorophores to barcode over 1,000 genotypes using a single (non‐iterative) hybridization. HiPR‐FISH was implemented to identify 1,023 distinct *E. coli* isolates across about 65,000 single cells.

*In situ* sequencing approaches also rely on transcribed perturbation barcodes (Fig 2A). Briefly, following fixation of cells, RNA barcodes are reverse transcribed to cDNA, and a padlock probe is used to copy the barcode into a circular single‐stranded DNA molecule, which serves as a template for rolling circle amplification (RCA) to clonally amplify the barcode sequence. Following amplification, barcodes are sequenced *in situ*, as demonstrated by several groups, with sequencing‐by‐synthesis (SBS) or sequencing‐by‐ligation chemistry (Fig 2C; Ke *et al*, 2013; Payne, 2017; Chen *et al*, 2018; Feldman *et al*, 2019). We describe the experimental procedure for ISS using SBS at length in a recent protocol publication (Feldman *et al*, 2022). In our initial demonstration, we studied 952 gene knockouts, measuring p65 localization in about 6 million cells in a series of screens and taking time course measurements of live cells for over 400,000 cells in targeted downstream screening (Feldman *et al*, 2019). Recently, we extended this approach to screen about 20,000 gRNAs targeting 5,072 essential genes, profiling DNA content, DNA damage, and microtubule and F‐actin subcellular organization across 31 million cells (Funk *et al*, 2022).

Lastly, in iterative IF approaches, perturbation barcodes encode unique protein epitope combinations that are transcribed and translated in live cells (Fig 2A; Wroblewska *et al*, 2018; Rovira‐Clavé *et al*, 2021). Following fixation, protein barcodes can be decoded through iterative IF measurements (Fig 2D). In an *in vivo* screen of 35 CRISPR KOs, Dhainaut *et al* (2022) used iterative IF to recover protein barcodes from about 1,750 tumor lesions in mouse tissue sections. By using protein barcode epitopes combinatorially, as many as 120 unique combinations have been demonstrated as distinguishable and greater barcoding complexity may be achievable by deploying additional orthogonal epitopes, higher order epitope combinations, and/or multiple barcodes with distinct subcellular localization (Wroblewska *et al*, 2018; Dhainaut *et al*, 2022; Kudo *et al*, 2022).

## Methods for genetic perturbation and barcoding

**Figure 3:**
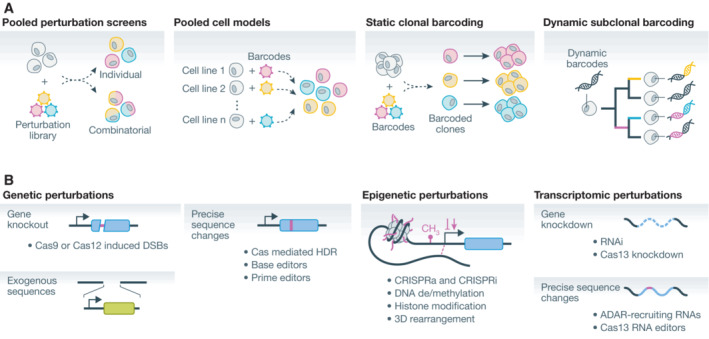
Cellular barcoding and perturbation (A) Applications of cellular barcoding. In pooled genetic screens, genetic perturbation libraries are used to introduce one or more genetic perturbations to cells with the same genetic background. Pooled cell models use barcodes to distinguish cells of different genetic backgrounds. Static clonal barcoding experiments use barcodes to track the progeny of individual clones. Dynamic subclonal barcoding approaches use dynamic barcodes to determine subclonal relationships between cells. (B) Approaches for programmable perturbation. Genetic perturbations, or changes to DNA sequence, include gene knockouts, introduction of new DNA sequences, or precise sequence changes. Epigenetic perturbations include changes to DNA accessibility, transcription factor recruitment, DNA methylation, histone modifications, and 3D genome structure. Transcriptomic perturbations include gene knockdown and precise sequence changes. DSBs, double strand breaks; HDR, homology‐directed repair; CRISPRa, CRISPR‐mediated activation of transcription; CRISPRi, CRISPR‐mediated interference of transcription; RNAi, RNA interference.

Pooled genetic screens are one of several approaches based on cellular barcoding, strategies that use molecular barcodes to enable recovery of information about cellular contents or identities (Fig 3A; Kebschull & Zador, 2018). These barcodes are most commonly DNA or RNA sequences read by sequencing at an experimental endpoint, though protein barcodes can be decoded with mass spectrometry or IF measurements (Wroblewska *et al*, 2018; Dhainaut *et al*, 2022). In the context of pooled genetic screens, cellular barcodes encode cellular genotype, for example, the gene target of a gRNA in a CRISPR KO screen. However, the applications of cellular barcoding extend beyond laboratory perturbations and engineered genetic differences that typify genetic screens.

In contrast to delivering a specific perturbation, pooled cell models leverage naturally occurring genetic and epigenetic diversity between cell models. In these approaches, cells barcoded according to their origin, such as cell lines from different individuals or tissue origins, can be assayed in a pooled setting. Pooled cell models have been used to identify anticancer reagents and characterize metastatic potential (Yu *et al*, 2016; Corsello *et al*, 2020; Jin *et al*, 2020). Cellular barcoding can also be used to track populations of cells over time. Static clonal barcoding is used to identify cells that originate from a single barcoded ancestor in a mixed population, and subclonal barcoding approaches use dynamic barcodes to identify subclonal relationships between cells (Kebschull & Zador, 2018). While this review is focused on genetic screening and the association of perturbation barcodes to resulting phenotypes, our discussion of methods to pair cellular barcodes to phenotypes is further relevant to this broader set of cellular barcoding methods.

In genetic screening approaches popular today, pooled cells differ in the sequence‐programmable perturbation each receives, and the perturbation sequence identifies the genotype of a cell (Fig 3A). Cells may contain a single perturbation or barcode, as in single gene knockout screens, or a combination or barcodes, as in the case of combinatorial perturbation screens. Technologies for programmable perturbation provide numerous ways to generate diversity in cell pools (Fig 3B). These include the introduction of exogenous DNA and perturbation of endogenous sequences by CRISPR. Further, we will consider analogous programmable methods that perturb properties of cells other than genomic sequence, such as RNA interference (RNAi) and RNA‐targeting CRISPR tools for transcriptomic perturbation and DNA‐binding CRISPR tools for epigenomic perturbation. Here, we discuss the diversity of methods for sequence‐barcoded perturbations and their applications in pooled screens.

### Targeted genomic perturbation screens with programmable reagents

RNAi and CRISPR offer powerful approaches for systematic genetic perturbation of user‐specified target sites in the genome, enabled by their straightforward sequence‐based programmability (Kennerdell & Carthew, 1998; Mohr *et al*, 2014; Shalem *et al*, 2015; Doench, 2018; Schuster *et al*, 2019). Today, this toolbox provides a diverse set of strategies to perturb the genome, epigenome, and transcriptome (Doench, 2018; Kampmann, 2018; Anzalone *et al*, 2020; Nakamura *et al*, 2021). Importantly for pooled approaches, the short hairpin RNA of RNAi and the gRNA of CRISPR are recoverable by sequencing after phenotypic profiling, and enable the identification of cellular genotype.

CRISPR KO was the earliest and is now the most widespread form of CRISPR perturbation used for genomic screening (Koike‐Yusa *et al*, 2014; Shalem *et al*, 2014; Wang *et al*, 2014). The first genome‐scale implementations of pooled CRISPR KO screens have identified genes involved in resistance to toxic pore‐forming and DNA‐damaging reagents (Koike‐Yusa *et al*, 2014), identified essential genes and genes involved in resistance to a therapeutic RAF inhibitor (Shalem *et al*, 2014), and revealed essential genes and genes involved in DNA repair pathways (Wang *et al*, 2014). Large‐scale CRISPR KO screens have since become a mainstay for mapping genotype–phenotype relationships. A number of reviews focus particularly on the design and execution of editing and delivery aspects of CRISPR KO screens (Shalem *et al*, 2015; Doench, 2018; Schuster *et al*, 2019).

In addition to gene knockouts, a variety of Cas enzymes are used to make precise edits in the genome (Anzalone *et al*, 2020). Some cells can be precisely modified through homology directed repair using an exogenous repair template encoding a user‐defined sequence change (Ran *et al*, 2013; Lin *et al*, 2014). Findlay *et al* (2014, 2018) used Cas9 with homology directed repair in pooled screens to characterize thousands of single nucleotide variants of BRCA1 and predict pathogenicity. The challenge of efficient and precise sequence modification also motivated the development of Cas fusion proteins capable of direct enzymatic alteration of DNA sequence (Komor *et al*, 2016; Gaudelli *et al*, 2017; Anzalone *et al*, 2019). Base editors—fusions of Cas enzymes to nucleoside deaminase domains—have been developed to effect nucleotide substitutions, primarily C‐to‐T and A‐to‐G transitions (Komor *et al*, 2016; Gaudelli *et al*, 2017) and employed in screens to profile sequence variants (Cuella‐Martin *et al*, 2021; Hanna *et al*, 2021).

Beyond DNA sequence modification, Cas enzymes are powerful tools for epigenomic perturbation. Nuclease‐inactive Cas enzymes, such as nuclease‐inactive “dead” Cas9 (dCas9), used essentially as programmable sequence‐specific DNA‐binding proteins, have been repurposed for interference (CRISPRi) and activation (CRISPRa) of transcription, achieving these outcomes through a diversity of mechanisms, most commonly through the recruitment of effectors (Kampmann, 2018; Nakamura *et al*, 2021). CRISPRa and CRISPRi approaches have since been employed in large‐scale screens (Gilbert *et al*, 2014; Konermann *et al*, 2015; Joung *et al*, 2017). The wide range of CRISPR‐based methods available for epigenome perturbation and their use in pooled screens have been reviewed elsewhere in detail (Kampmann, 2018; Schuster *et al*, 2019; Nakamura *et al*, 2021).

Finally, RNAi and CRISPR both enable targeted transcriptomic perturbations. Genome scale RNAi screens are a well‐established method to systematically perform RNA knockdown (Mohr *et al*, 2014). Cas13‐based RNA knockdown methods have been developed more recently and may offer advantages in knockdown specificity, though they are rarely applied for screening (Abudayyeh *et al*, 2017; Wessels *et al*, 2020).

### Exogenous sequences

The introduction of exogenous regulatory or coding sequences is another approach to perturb genotype. Such approaches have been used to study the function of noncoding sequences, sequence variants, or gene function in a non‐native context. In massively parallel reporter assays, noncoding sequences are placed upstream of a reporter gene to screen for gene regulatory functions in isolation from their genomic context (Patwardhan *et al*, 2009, 2012; Kinney *et al*, 2010). Screens of sequence variants can reveal the impact of sequence changes to proteins or noncoding regions. Sequence variants can be generated with genome editing, to modify an endogenous gene and maintain endogenous regulation, or delivered as exogenous *in vitro* synthesized DNA or RNA. Interrogating the functional significance of genetic variation is one such application, with optical readouts providing the opportunity to assay multiple aspects of gene function, such as subcellular localization and colocalization with relevant factors. For example, Hasle *et al* (2020) screened a library of simian virus 40 nuclear localization signal mutants, using an image‐based enrichment step to identify variants impacting nuclear translocation of a fluorescent reporter. And morphological profiling has been used to screen genetic variants and cluster variants by phenotype (Rohban *et al*, 2017; Caicedo *et al*, 2022). Lastly, both CRISPRa and exogenous sequence expression can enable the study of genes that are transcriptionally inactive in the chosen cell model, with these two approaches providing consistent or complementary information depending on the context (Sanson *et al*, 2018).

### Combinatorial approaches

Pooled screens with single‐cell readouts offer an attractive format for the implementation of combinatorial screening approaches. Two challenges arise in conducting combinatorial screens, relative to traditional screens. The first challenge is the size of the combinatorial space; a screen of pairs among just 200 genes is comparable in scale to a typical genome‐wide screen. The scalability of pooled approaches offers an important advantage toward the practicality of combinatorial screens. The second challenge is the readout of perturbation co‐occurrence. Combinatorial screens with standard perturbation reagents are compatible with pooled profiling but enrichment screens are not. While enrichment screens rely on perturbation barcode abundance and cannot maintain linkage between paired perturbations, profiling screens genotype perturbations in individual cells, revealing co‐occurrence of multiple perturbations at the cellular level and matching these to single‐cell phenotypes. However, specialized perturbation library designs physically link multiple perturbations, such as an array of gRNAs, or employ an auxiliary barcode unique to a perturbation combination, enable compatibility with enrichment screens (Wong *et al*, 2016; Erard *et al*, 2017; Shen *et al*, 2017; Zhou *et al*, 2020). In these approaches, Cas12 nucleases are an attractive alternative to Cas9 for their capacity to process gRNA arrays into active individual gRNAs (Fonfara *et al*, 2016; Zetsche *et al*, 2017). While elegant and capable of delivering a specific set of combinations in a single step with a single selection, these approaches require dedicated library synthesis. Approaches that rely on grouping multiple active or proxy encoding elements (e.g., gRNAs, barcodes) face additional challenges in maintaining linkage to the end of the workflow due to the possibility of recombination, particularly when linked elements are widely separated (preprint: Feldman *et al*, 2018).

### Delivery

All the methods discussed above for generating or tracking cellular diversity require the delivery of barcoding/perturbation reagents. It is a requirement that cells maintain a copy of the perturbation reagent/barcode for the duration of the experiment to enable endpoint recovery of perturbation genotypes. Lentiviral systems are the most well‐established delivery method for pooled RNAi and CRISPR screens. These genome‐integrating vectors are routinely applied to produce high‐complexity, high‐titer libraries and are capable of transducing many cell types at high efficiencies, including some non‐dividing cells (Yip, 2020). Vector transduction at low multiplicity of infection can ensure that most cells receive a single perturbation. In CRISPR‐based approaches, delivery of both a gRNA and Cas enzyme are required; however, because only the gRNA sequence needs to be recovered for genotyping, Cas enzymes may be expressed continuously or delivered transiently (Shifrut *et al*, 2018). Adeno‐associated viruses (AAVs) are a useful alternative to lentiviruses for efficient *in vivo* delivery of perturbation reagents (Chow *et al*, 2017; Wang *et al*, 2018a). While AAVs are non‐integrating, they have been engineered to encode a transposon capable of genomic integration, enabling pooled *in vivo* CRISPR screens (Ye *et al*, 2019). Similar strategies may co‐opt other non‐integrating viruses for delivery of pooled screening reagents. Transfection of transposon‐based genome‐integrating reagents have also been employed as an alternative to viral delivery (Li *et al*, 2017; Xu *et al*, 2017; Viswanatha *et al*, 2018).

## Optical methods for multidimensional phenotypic profiling

**Figure 4:**
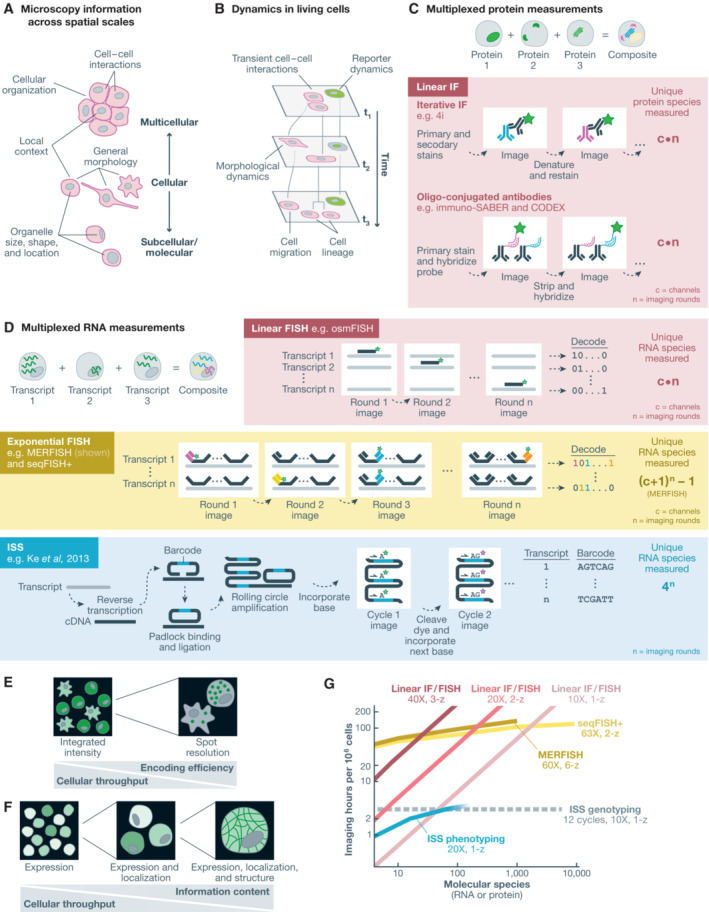
Optical methods for multidimensional phenotypic profiling (A) Microscopy can capture molecular, subcellular, cellular, and multicellular phenotypes. (B) Live cell microscopy captures cellular dynamics. (C) Multiplexed protein measurements enable the observation of multiple proteins in each cell. Iterative immunofluorescence approaches rely on multiple rounds of sample staining with dye‐conjugated antibodies and fluorescence imaging. Oligo‐conjugated antibodies enable measurement with hybridization of fluorescence *in situ* hybridization probes. (D) Multiplexed RNA measurements enable the observation of multiple RNA species in each cell through either fluorescence *in situ* hybridization (FISH) or *in situ* sequencing (ISS). Linear FISH approaches encode RNA measurements with a linear encoding relative to imaging iterations. Exponential FISH techniques facilitate measurement of an exponentially increasing number of RNA species with a linear increase in imaging iterations. ISS approaches similarly enable exponential encoding of RNA species across sequencing cycles. Encoding efficiencies are theoretical and do not account for additional imaging cycles/rounds used for error correction in exponential‐scaling techniques. (E) RNA measurements requiring spot resolution can efficiently encode many RNA species but require high‐magnification imaging, while linear encoding methods can rely on integrated intensity measurements, instead of spot resolution, to enable high throughput at lower optical magnification. (F) Spatial detail and imaging time both increase as protein localization is imaged at higher magnification, presenting a tradeoff between cellular throughput and information content. (G) Estimated imaging throughputs and multiplexing capacities for selected multiplexed RNA and protein measurement approaches, based on magnification and z‐stack requirements, fluorescence channels, imaging rounds, and theoretical encoding efficiency without error correction. For comparison, the required imaging time to genotyping one million cells with 12 cycles of *in situ* sequencing is shown in gray. At high multiplexes, ISS phenotyping becomes less quantitative due to optical crowding. We do not consider expansion microscopy approaches here, which can increase the maximum multiplex for resolution‐limited techniques at the cost of increased imaging time. IF, immunofluorescence; FISH, fluorescence *in situ* hybridization; ISS, *in situ* sequencing.

Microscopy is a high‐dimensional profiling tool that inherently provides optical measurements at subcellular, cellular, and multicellular resolutions simultaneously (Fig 4A). For example, a single image of thousands of cells can capture cell size and shape, subcellular characteristics, such as the size, shape, and location of organelles, and the juxtaposition of cells in a multicellular setting, such as in a natural or engineered tissue (Boutros *et al*, 2015). And microscopy is one of few techniques that straightforwardly enables dynamic multiplexed measurements of single cells today. Live cell imaging extends features at all levels of spatial resolution as time series replete with kinetic information about how data features change over time at steady state and/or in response to perturbations (Fig 4B). The combination of microscopy with molecular measurements of DNA, RNA, and proteins has expanded the phenotypic dimensions accessible to optical methods and made microscopy images more directly relatable to molecular quantification data. Some, though not all microscopy techniques and molecular imaging methods are compatible with live‐cell microscopy and imaging of living tissue samples.

Fundamentally, biological images of cells are exceedingly high‐dimensional, with the dimensionality even exceeding the product of pixel count per cell and number of markers or channels as combinations of pixel/marker values and pixel/pixel correlations within and across cells often reflect phenomena of interest. For example, pixel‐pixel correlations may report the ruffling of a membrane structure or juxtaposition of different cells. Similarly, marker‐marker correlations in a single pixel may report co‐localization of proteins or other molecular species (Gut *et al*, 2018).

We will provide an overview of the diverse suite of methods for multiplexed molecular measurements with microscopy with a focus on the tradeoffs between cellular throughput and phenotypic dimensionality. These approaches are often compared to non‐spatially resolved single‐cell “omics” methods, such as those based on sequencing or CyTOF. Several such methods can be practiced with relative experimental ease, though at the cost of rich spatial information and/or scale. We will limit detailed discussion to imaging‐based methods proven for high‐throughput implementation or with such potential.

### Live‐cell imaging

Live‐cell microscopy enables the direct observation of individual cell behavior, such as morphological dynamics, responses to stimuli, motility, transient cell–cell interactions, and cell division events and lineage relationships (Fig 4B). Combined with two major advances of past decades—recombinant DNA and the discovery of fluorescent proteins—time‐lapse fluorescence microscopy has also illuminated molecular dynamics of living cells. The fusion of a fluorescent protein to a target protein enables visualization of the expression and localization of the target protein over time. An assortment of tools for fluorescent tagging of RNA in live cells have also been developed and have been discussed at length elsewhere (Specht *et al*, 2017; Pichon *et al*, 2018). The extent to which such live‐cell molecular imaging approaches can be multiplexed is limited by the requirement for cell line engineering and the spectral bandwidth of the reporters. Despite this limitation, live‐cell imaging is a powerful single‐cell technique capable of capturing a high‐resolution temporal dimension inaccessible to most other approaches.

In two examples, Schmitz *et al* (2010) and our group leveraged live‐cell imaging in screens to study the dynamic events of mitosis (Funk *et al*, 2022). Schmitz *et al* (2010) identified regulators of mitotic exit through an arrayed RNAi screen with live‐cell imaging, tracking individual cells with nuclear and histone fluorescent reporters. A decade later, our group used a nuclear stain to perform a live‐cell pooled CRISPR KO screen as part of a study identifying essential genes that altered mitotic frequency and duration (Funk *et al*, 2022).

### Protein and organelle tools

Visualization of subcellular structures and proteins in fixed samples is enabled by a range of small molecule and antibody‐based approaches. We will highlight a few illustrative examples, and these methods have recently been reviewed in greater detail elsewhere (Tan *et al*, 2020; Hickey *et al*, 2021; Lewis *et al*, 2021; Moffitt *et al*, 2022). Fluorescence microscopy‐based approaches include IF and small molecule stains that can be straightforwardly combined up to several‐plex across standard fluorescence imaging channels. The Cell Painting method is one approach that enables visualization of major cellular compartments including the nucleus, nucleoli, endoplasmic reticulum, Golgi, mitochondria, actin, cytoplasmic RNA, and plasma membrane via six dyes in five fluorescence channels. Cell Painting enables measurement of thousands of raw features and hundreds of orthogonal features at single cell resolution (Bray *et al*, 2016). Methods for reversible antibody staining have enabled IF multiplexing beyond the number of fluorescence channels, through iterative staining, imaging, and destaining (Fig 4C). A method termed 4i uses traditional IF staining and imaging followed by a denaturation‐based signal removal (Gut *et al*, 2018). 4i has been demonstrated for multiplexing of up to 40 antibody stains in images of hundreds of thousands of cells. Immuno‐SABER is another cyclic method and uses oligo‐conjugated antibodies with DNA hybridization‐based amplification for visualization that has been demonstrated up to 10‐plex (Saka *et al*, 2019). Additional approaches include multiplexed ion beam imaging (MIBI), which uses highly spatially resolved time‐of‐flight mass spectrometry to resolve metal‐tagged antibodies, and digital spatial profiling (DSP), which uses photocleavable oligo‐antibody conjugates that can be selectively released from regions of a sample for sequencing (Keren *et al*, 2019; Merritt *et al*, 2020). Mass spectrometry and NGS‐linked approaches each offer distinct advantages but do not currently meet the requirement of high cellular throughput with single‐cell resolution necessary for large‐scale pooled genetic screening. Because fluorescence methods are accessible using commonly available fluorescence microscopes, are relatively fast and affordable, and have already been employed at scale, these approaches are the most immediately promising for high‐throughput profiling of subcellular structure with pooled genetic perturbations in a large number of laboratories.

### RNA tools

Fluorescence *in situ* hybridization and ISS approaches have enabled multiplexed measurements of nucleic acids at cellular and subcellular spatial resolutions for the assessment of transcriptional activity as a phenotypic screening readout (Fig 4D). Iterative imaging‐based methods rely on serial probe hybridization or sequencing cycles—both approaches requiring the signal from individual RNA or cDNA molecules to be detected and processed separately. RNA measurement techniques achieve transcript multiplexing through a combination of spatial, spectral, and temporal separation. However, each of these factors is inversely related to the cellular throughput of the method. Spatial resolution—resolution to distinguish adjacent spots to enable the measurement of distinct transcripts and species in space—is linked to magnification, which in turn is related to imaging time in proportion to its square. Magnification is also an important parameter with respect to signal‐to‐background ratio, with higher magnification enabling higher signal‐to‐background ratio, but also reducing depth‐of‐field, possibly requiring additional z‐stacks and further increasing imaging time. Signal amplification techniques, including RNAscope, branched DNA amplification, ClampFISH, HCR, SABER, and RCA can increase signal over background reducing light‐collection/magnification requirements, though requiring increased chemistry time and potentially decreasing achievable spatial resolution by increasing spot sizes (Stougaard *et al*, 2007; Choi *et al*, 2018; Kishi *et al*, 2019; Rouhanifard *et al*, 2019; Xia *et al*, 2019a). Notably, while several super‐resolution techniques can refine the position of signal centers in individual images where signal‐generating molecules are sparse, these are not useful for segregating crowded signals that overlap in the temporal and spectral dimensions.

Spectral separation entails splitting signals across fluorescence channels. When channels are imaged one at a time (as opposed to hyperspectral imaging that detects and distinguishes multiple colors at once; Shi *et al*, 2020), imaging time scales linearly with the number of fluorescence channels.

Lastly, temporal separation is used to encode information across multiple rounds of imaging. To separate signals across imaging rounds, chemistry steps are performed between rounds of imaging, removing signals from the previous round and generating new signals for the next round. Temporal separation has been used to computationally reconstruct subdiffraction limit information in a subset of super‐resolution imaging techniques (Eng *et al*, 2019). Total sample processing time in temporal separation protocols depends on the chemistry time, imaging time per round, and number of rounds. For fast imaging times or slow chemistry steps, chemistry time can be a major contributor to overall throughput, though parallelization of imaging and chemistry steps on multiple samples generally negates this impact for large projects.

For each category, we again provide demonstrative examples for these technologies while referring readers to other reviews for more details (Lewis *et al*, 2021; Rao *et al*, 2021; Moffitt *et al*, 2022). FISH is a long‐established technique for detection of RNA species in fixed tissues. Linear‐scaling or “linear” FISH methods encode one RNA species per fluorescence channel and imaging round. One such method, osmFISH, quantified 33 RNA species in about 5,000 cells through 13 sequential imaging rounds in three fluorescence channels (Codeluppi *et al*, 2018). Exponential‐scaling or “exponential” FISH techniques have enabled quantification and localization of dozens up to as many as 10,000 transcripts using sequence encoding schemes and sequential FISH measurements. The approaches seqFISH+ and MERFISH have demonstrated the greatest multiplexing capabilities, each enabling measurements of up to 10,000 transcripts and have been demonstrated at a throughput of a few thousand cells (Eng *et al*, 2019; Xia *et al*, 2019b). These highly multiplexed techniques require high‐sensitivity and high (super‐) resolution imaging that limit field size and cellular throughput. An important distinction between linear and exponential FISH approaches is the requirement for spot resolution (Fig 3E). Exponential FISH methods rely on the digital decoding of individual spots over multiple fluorescence channels and cycles to decode RNA species, such that multiplexing capacity and quantitation are dependent on high‐resolution imaging. In contrast, linear FISH approaches do not require spot resolution and can instead use graded cell‐level metrics such as integrated intensity, enabling imaging across larger fields at lower spatial resolution. FISH signal amplification techniques are also important to enable sufficient signal intensity for large‐field imaging with lower collection efficiency optics.

Padlock probe/ISS approaches offer moderate multiplexing capacity at high cellular throughput (Ke *et al*, 2013; Lee *et al*, 2014; Qian *et al*, 2020; Alon *et al*, 2021). ISS techniques for transcript localization and enumeration are performed analogously to ISS genotyping methods (Fig 2C). Direct ligation of padlock probes on RNA or cDNA targets and detection by hybridization to a target‐linked sequence in the amplified padlock enable simplified workflow for multiplexed analyses of transcription. Even so, multiple sequencing chemistries including sequencing‐by‐synthesis and sequencing‐by‐ligation have been demonstrated for ISS applications. For example, the pci‐Seq approach uses barcoded padlock probes to measure 99 transcripts by ISS by ligation and achieved cell‐type identification in approximately 27,000 mouse hippocampal cells (Qian *et al*, 2020). In contrast to exponential FISH approaches that require higher magnification and greater temporal separation, ISS approaches enable exponential encoding at relatively high cellular throughput. However, ISS approaches still require spot resolution and optical crowding ultimately limits ISS to lower maximum readout bandwidth per cell. Taken to an extreme, FISSEQ uses ISS to make untargeted measurements of RNA in cells (Lee *et al*, 2014). FISSEQ measurements have been limited to detecting about 200 spots corresponding to mRNA transcripts per cell in primary human fibroblasts (Lee *et al*, 2015). While all exponential multi‐cycle multiplexing methods entail necessary compromises across multiplexing capacity, quantitative dynamic range, cellular throughput, ISS approaches quickly lose quantitative accuracy in the face of signal crowding (Lee *et al*, 2014; Qian *et al*, 2020). One solution to increasing the multiplexing capacity of ISS approaches is targeting panels of orthogonally expressed transcripts, reducing spot density within cells while maintaining high biological information content. In the STARmap ISS approach, a 1,020‐transcript panel was designed such that a distinct subset of the panel was expected to be expressed in each of about a dozen distinct cell types (Wang *et al*, 2018c).

Spatially barcoded oligo arrays provide a distinct path to link image‐based phenotyping with genotyping—using NGS. These approaches use arrays of oligos with spatial barcodes that identify their location; overlaying a thin biological sample on the array, these oligos capture cellular RNA for measurement with NGS, after which sequences are mapped to positions using the spatial barcodes (Ståhl *et al*, 2016; Rodriques *et al*, 2019; Vickovic *et al*, 2019; preprint: Fu *et al*, 2021; Stickels *et al*, 2021). These approaches offer straightforward high‐plex and spatially‐resolved transcriptomic measurements via standard RNA‐seq without a demanding optical microscopy requirement. However, while transcript capture rates and spatial resolution are improving, the capture efficiency remains well below that of FISH approaches (Grün *et al*, 2014; Stickels *et al*, 2021). Further, assigning reads to individual single cells—typically essential to pooled screening—remains challenging, and throughput is limited by the size of available arrays and sequencing costs. In a notable exception, spatially localized clonal populations, such as tumor lesions, are sufficiently large to enable clonal, but not single‐cell, measurements with current spatially barcoded oligo arrays (Dhainaut *et al*, 2022). As these technologies continue to mature to enable reliable molecular‐cellular assignments, arrays could be an excellent approach for optical and transcriptional readout of *in vivo* screens in tissue sections with densely packed cells.

### DNA tools

Methods analogous to spatial transcriptomics have been developed for measurements of DNA in fixed cells for the determination of 3D genome organization (Payne *et al*, 2021; Takei *et al*, 2021). These methods have recently been reviewed in greater detail elsewhere (Xie & Liu, 2021). DNA seqFISH+, a sequential DNA FISH technique, demonstrated 3,660 assessments of DNA locus position across the genome (Takei *et al*, 2021). And *in situ* genome sequencing (IGS), an approach that pairs ISS and NGS, enabled hundreds to several thousand measurements of genomic loci in individual cells (Payne *et al*, 2021). Like morphology, protein organization, or transcriptome, 3D genome organization is a representation of cell phenotype that could be valuable to probe in a screening context. For example, such a screen could systematically profile the role of putative CTCF binding sites in maintaining genome structure and gene activity. While these approaches have impressive multiplexing capabilities, demonstrations of both DNA seqFISH+ and IGS have been limited to a few hundred cells, owing to the requirement for high‐magnification imaging and many rounds of FISH or ISS chemistry. Even so, alternative implementations of DNA seqFISH+ and IGS at lower multiplexing capacity might be amenable to higher cellular throughput to support genetic screens.

### Other approaches

While optical approaches preserving spatial information are the focus of this review, it is worth briefly acknowledging the wide range of non‐optical approaches for multidimensional profiling of single cells (Spitzer & Nolan, 2016; Kashima *et al*, 2020). scRNA‐seq and scATAC‐seq are now routine procedures with commercially available reagents, and many more approaches have been developed to probe the genome, epigenome, and transcriptome at single‐cell resolution. Several groups have integrated these sequence‐based readouts with CRISPR‐based perturbation in screening contexts (Adamson *et al*, 2016; Dixit *et al*, 2016; Jaitin *et al*, 2016; Datlinger *et al*, 2017; Rubin *et al*, 2019; Replogle *et al*, 2020). The throughput of these approaches is primarily limited by consumables and sequencing costs, though large‐scale screens are possible (Replogle *et al*, 2022). Flow cytometry‐based approaches are common for single‐cell proteomics, including CyTOF, which enables multiplexed measurements of up to about 40 proteins, and can be used for pooled profiling screens with protein barcodes (Wroblewska *et al*, 2018; Ijsselsteijn *et al*, 2019; Dhainaut *et al*, 2022). These approaches enable a parallel set of applications within the accessible scales and share aspects of experimental design and analysis as optical techniques. We expect such approaches to provide information that is highly complementary to optical readouts and in some cases be convenient to run in tandem (e.g., scRNA‐seq and optical pooled screens with the same CROP‐seq cell library). As microscopy, cytometry, and sequencing based screens are used together, it is important to explore how these technologically distinct measurements can most effectively combine to provide complementary information.

## Analysis methods

The analysis of screening data is a critical step in the extraction of biologically meaningful insights. Generally speaking, enrichment screen analysis approaches are similar across enrichment methods, simplified by the standardized output of barcode counts generated from next generation sequencing data. Many tools for the downstream analysis of enrichment screens have been developed and reviewed elsewhere (Lin *et al*, 2020). Microscopy‐based profiling screens present two specific analysis challenges, (i) primary image analysis: the extraction of information from images including cell segmentation, perturbation barcode assignment, and phenotypic feature extraction; and (ii) secondary analysis: classification, testing, and interpretation of multidimensional single‐cell phenotypes based on extracted phenotypic features.

### Primary image analysis

Primary image analysis, the raw‐pixel‐facing initial step, is complex to solve at a general level because appropriate image analysis approaches and performance depends to varying degrees on sample type, assay type, and sample processing/image acquisition conditions. In contrast to the production and analysis of digital NGS data counts, image acquisition and analysis methods are not standardized, instead requiring significant tailoring for specific screening projects. As a limited exception, we find that genotyping by ISS using our protocol can be largely standardized as a common set of reagents, conditions, and analytical steps for ISS are broadly applicable and produce digital sequence counts for each cell. Even so, some sample and protocol‐dependent aspects of image analysis—like cell segmentation—apply to both phenotyping and genotyping.

Cell segmentation illustrates a common performance‐generalization tradeoff common to image‐based analysis. Ideal cell segmentation is a difficult task, but segmenting nuclei is straightforward when data from a nuclear marker (e.g., DAPI) are available. Nuclear segmentations are highly generalizable, but restricting ISS genotyping or phenotype feature extraction to analysis of pixels overlapping nuclei represents a performance compromise as a lower fraction of cells will receive genotype assignments and the set of accessible phenotypes will be restricted. Deep learning‐based approaches, such as Cellpose, have been demonstrated to perform accurate cell segmentation on diverse sample types and may provide a more flexible segmentation solution that requires less user expertise and fine‐tuning, albeit at increased computational expense (Stringer *et al*, 2021). The single‐cell‐centric analysis paradigm in profiling screens simplifies image analysis requirements in some ways. For example, there is no need for precise alignment of pixel‐level results from genotyping and phenotyping datasets if cell genotypes and cell phenotypes are each referenced to a common set of segmented cellular indices established using a gross alignment. This is particularly advantageous when a substantially different microscope configuration or technique is desired for cellular phenotyping.

Many algorithms exist to extract phenotypic features from segmented cell images. CellProfiler is an open‐source suite that incorporates many tools for extraction of thousands of predefined features from cell images (Carpenter *et al*, 2006; Dao *et al*, 2016; McQuin *et al*, 2018). In addition, supervised and unsupervised machine learning methods have been developed to computationally identify image features that “predict” cell class labels or efficiently characterize variability present in a dataset (Shifat‐E‐Rabbi *et al*, 2020). Addressing the challenges in primary image analysis requires user‐friendly, robust, and high‐throughput image analysis methodologies that flexibly and intelligently incorporate known biological context.

### Secondary analysis

Approaches to interpreting multidimensional single‐cell phenotypes have begun to emerge in the context of analysis of single‐cell sequencing‐based profiling screens, though their implementation in image‐based screens with optical phenotypic features is at an earlier state of development (Dixit *et al*, 2016; Duan *et al*, 2019; Norman *et al*, 2019; Yang *et al*, 2020; Wang, 2021). The rich data (cells by perturbations by features) obtained from profiling screens can be mapped to the traditional enrichment screening workflow as a large set of univariate screens based on a central tendency statistic (e.g., median across cells) of individual features. A drawback is the necessity to carry out a multiple hypothesis testing correction, which is exacerbated as additional metrics such as feature combinations (e.g., ratios) are appended. Naturally, in single cell‐resolved pooled profiling data, there is a further opportunity to test for distributional effects observed across cells in scored criteria.

True multidimensional analysis of large datasets is an active area of research in computational biology, and incorporating cell‐level distribution data is at the cutting edge today. Much of the activity has focused on the explosion of available RNA‐seq and scRNA‐seq data, with pooled screens using scRNA‐seq or ATAC‐seq readout providing data matrices structured similarly to single‐cell resolved image datasets (cells by perturbations by features). However, molecular “omic” data features are simply—if incompletely—contextualized by genomic annotation, allowing rapid initial interpretation. While the ultimate basis of image features in cell biology represents a tremendous opportunity, conceptual frameworks and methods for mapping of these effectively analog, resolution‐ and marker‐dependent signals into spatially‐ and molecularly‐resolved cell biology are complex and at an early stage of development. While analysis of morphological data is a ripe area for further development, some useful approaches are emerging from the broader single‐cell analysis playbook.

A starting point useful for many projects is image feature selection based on the statistics of features across the comparison set followed by regularization and dimensional reduction using one of a variety of machine learning approaches including principal components analysis, pre‐trained convolutional neural networks (CNNs), or novel CNNs generated using an autoencoder. Results may be visualized for exploration using one of a variety of 2D embedding techniques (UMAP, t‐SNE) and clustering approaches (hierarchical, dbSCAN, Leiden, Louvein) at varying levels of clustering resolution and overlaid annotation of perturbation identities/categories. A set of control perturbations with previously interpreted biological effects of interest in the system serve as key reference points to guide such analyses. For example, variability in observed phenotypes across multiple perturbations of the same control gene or control genes with related functions provide measures of variability. Considering the clustering results together with the spread of control genes and the biological annotations/priors of all genes is a useful basis for initial interpretation of multiparameter results. From there, external data reflecting gene–gene relationships can be overlaid to further aid biological interpretation, and individual feature scores can be overplotted to discover which particular image features are driving plotted variability of biological import.

## Roadmap for future methods

The future of optical methods for pooled genomic profiling screens will see the integration of many of the experimental approaches we have discussed. In this section, we propose a roadmap for the integration of new perturbation modalities and phenotypic measurements, the extension of approaches to new biological model systems, and improvements in workflows to enable greater scale and dissemination of technologies. While some aspects we discuss apply to the broader set of microscopy‐based screening technologies, we focus our discussion on future implementations of pooled profiling technologies, in particular, ISS‐based optical pooled profiling screens of mammalian cells (Feldman *et al*, 2019, 2022; Funk *et al*, 2022).

### Perturbation modalities

Emerging perturbation technologies promise to make new perturbation modes accessible to screens. Prime editors, fusions of Cas9 to a reverse transcriptase, have been demonstrated to programmably write insertion, substitution, and deletion mutations (Anzalone *et al*, 2019; preprint: Ioannidi *et al*, 2021). In particular, screens of naturally occurring genetic variants, the installation of which often requires greater precision and flexibility than other approaches enable, would benefit from the application of prime editors to screens (Erwood *et al*, 2022). For epigenetic perturbation, fusions of Cas9 to enzymatic domains have enabled modulation of DNA methylation and histone methylation and acetylation (Hilton *et al*, 2015; Kearns *et al*, 2015; Polstein *et al*, 2015; Thakore *et al*, 2015; Choudhury *et al*, 2016; Vojta *et al*, 2016; Kwon *et al*, 2017). Implemented in screens, these approaches would provide a greater diversity of perturbations to regulation—targeting range, effect duration, effect magnitude, dependency on basal epigenetic state—than the CRISPRi and CRISPRa tools currently employed in screens. RNA‐guided Cas protein fusions have even been leveraged to alter 3D genome organization and the juxtaposition of genomic loci with liquid–liquid phase segregated domains (Wang *et al*, 2018b). Expanded to screening contexts, such technologies could systematically dissect the impact of specific genomic/cellular organizational paradigms, genomic interactions, and gene regulatory paradigms.

The extension of new programmable perturbation approaches to screening contexts first relies on a robust understanding of the performance of these technologies to enable effective *in silico* experimental design. For example, genetic knockout screens with Cas enzymes have undergone optimizations for nuclease expression and nuclear localization as well as guide RNA selection for on‐target activity, including for achieving specific DNA repair outcomes, and minimizing the risk of off‐target edits (Doench *et al*, 2014, 2016; Henkel *et al*, 2020; Michlits *et al*, 2020; DeWeirdt *et al*, 2021). These efforts have yielded ready‐to‐use Cas9 constructs and validated guide RNA libraries for cell lines from multiple species. Similar optimizations will be required to bring precision sequence changes and the wide array of programmable epigenetic perturbation approaches to widespread use in screening contexts. Significantly, profile‐based screening on highly scalable platforms may not require the same level of perturbation penetrance as enrichment screens, speeding adoption of new perturbation approaches. Single‐cell‐level readout of complex profiles can in principle enable sensitive filtering of perturbed versus non‐perturbed cells to select a perturbed cell set for analysis with enhanced statistical power. Where throughput is high enough to sample many cells per perturbation, screening may be practical and effective even when the fraction of perturbed cells is low.

Combinatorial optical pooled screens offer exciting opportunities to explore genetic interactions. Additionally, concepts from compressed sensing may decrease the requisite cellular throughput of screens exploring large combinatorial spaces (preprint: Cleary & Regev, 2020). The detection of multiple perturbation barcodes per cell is the primary technical hurdle in implementing combinatorial optical pooled screens. The design of perturbation reagents encoding multiple perturbations arrayed on a single transcript, such as Cas12 gRNA arrays, and optionally associated with an auxiliary barcode offer one solution (Wong *et al*, 2016; Erard *et al*, 2017; Shen *et al*, 2017; Zhou *et al*, 2020). Alternatively, with a single‐cell readout, pooled profiling screens can recover the identity of multiple single‐perturbation reagents or barcodes delivered to cells. This approach may be immediately accessible in some systems utilizing existing single‐perturbation libraries delivered serially or at multiplicity to cells.

### Multiplexed molecular measurements

The diverse array of multiplexed *in situ* proteomics and transcriptomics techniques promise integration of these information rich measurement modalities with microscopy‐based pooled profiling screens. The primary challenge in integrating these approaches with screens will be adapting profiling technologies—typically designed for maximal multiplexing capacity rather than speed—to meet the cellular throughput requirements of screens. As demonstrated, pooled profiling screens already integrate multiplexed protein (IF) and RNA (sgRNA genotyping by ISS) measurements, and the future for screens with flexibly integrated multimodal *in situ* measurements of native proteins and transcripts at higher multiplex is bright.

As protein localization measurements in genetic screens are uniquely accessible to microscopy‐based screening approaches, the integration of multiplexed protein measurements with optical pooled screens is particularly attractive. In contrast to RNA measurements which are often interpreted as a proxy for protein expression level, protein measurements can reveal directly interpretable functional molecular features, such as post‐translational modifications, subcellular localization (RNA localization is generally less functionally impactful), molecular interactions, and intricate cellular structures with known functions. Thus, while each RNA abundance measurement provides a single phenotypic dimension in most use cases, imaging even a single protein with subcellular and/or temporal resolution yields multidimensional phenotypic information. This is an important distinction to note when we compare the multiplexing capacities of RNA and protein measurement approaches; while the dimensionality of RNA measurements can be considered equivalent to the degree of multiplex, the effective dimensionality of protein measurements may commonly be a factor higher than the degree of multiplex.

In terms of protocol compatibility and throughput, linear scaling iterative IF techniques are attractive methods to integrate with optical pooled screens. Protein multiplexing limits with IF are bound by the availability of quality affinity reagents, the spectral properties of fluorescent labels, the spectral performance of the imaging system, and the time required for imaging and chemistry steps. In terms of capacity to detect a given protein or particular post‐translational modification, the availability and identification of quality antibody reagents is the major limiting factor today, and there is a clear need to expand the catalog of affinity reagents specific to modified peptide sequences that can efficiently penetrate sample matrices. Techniques that use chemical means to remove signal between rounds, rather than photobleaching, are generally favorable due to overall speed for large samples and the ability to parallelize imaging and signal removal by removing samples from the optical system for de‐staining operations. Within chemistry‐based multiplexing methods, while minimizing incubation times is favorable, parallelization can mitigate the impact of incubation time for large samples. In particular, approaches like 4i, Immuno‐SABER, and CODEX appear attractive for multiplexing tens of protein targets at high cellular throughput (Goltsev *et al*, 2018; Gut *et al*, 2018; Saka *et al*, 2019). We expect integrating these approaches into the existing optical pooled screening genotyping procedure to be relatively straightforward, as we have already implemented one reversible IF protocol developed at our institute with optical pooled screens (Funk *et al*, 2022), although extensive staining, imaging, and destaining may pose challenges for sample stability and feature fidelity at the highest levels of multiplexing.

For protein measurements, there is a tradeoff between information content and throughput. For simple abundance measurements, the lowest tolerable resolution is desirable for maximal throughput. However, greater lateral and z‐imaging resolution may reveal important subcellular details not otherwise discernable (Fig 4F). Where there is an *a priori* phenotype of particular interest, the optimal imaging configuration will be the lowest magnification and fewest z‐stacks that can be validated to resolve that phenotype. Otherwise, for profiling studies, the maximum optical resolution achievable in a tolerable imaging time is advisable to maximize useful feature yield and phenotyping power. Considering the demonstrated throughput of multiplexed IF techniques and estimated magnification‐dependent imaging times, multiplexed measurements of up to 40 protein species should be within reach of high‐throughput screens at 10× magnification, while higher magnifications may be tolerable at lower multiplexes (Fig 4G).

Considering the multiplexing and throughput tradeoffs of RNA measurement techniques, measurements of tens to hundreds of transcripts, but not thousands, are already feasible for screens of millions of cells (Fig 4G). Targeted ISS of transcripts is one approach to measure a select number of RNA species, using encoding schemes that enable measurement of up to 4^n^ RNA species over n imaging rounds. Further, this approach would be “natively compatible” with ISS‐based perturbation barcode sequencing in optical pooled screens. Given implementation with current protocols at 20× magnification, we expect that the upper multiplexing limit for quantitative measurement will be fewer than 100 RNA species today (Lee *et al*, 2014, 2015; Qian *et al*, 2020).

Linear scaling FISH approaches are another attractive approach that compensate for lower encoding power with low‐magnification imaging capability. Similar to protein measurements, imaging times will likely limit linear FISH multiplexing to tens of transcripts (Fig 4G). Composite RNA measurements and computational signal separation using compressed sensing approaches offer one potential solution to expand the capacity of linear FISH approaches beyond apparent multiplexing limits defined by the product of fluorescent channels and imaging rounds (Cleary *et al*, 2017, 2021). Lastly, owing to the requirements for high spatial resolution, transcriptome‐scale methods like seqFISH+ and MERFISH are currently suited to screens of tens of thousands of cells or fewer and indicated when a small number of perturbations are of *a priori* interest or nominated from other large‐scale screening results (Eng *et al*, 2019; Wang *et al*, 2019; Xia *et al*, 2019b; Fig 4G).

Additional considerations to throughput not yet addressed here are the cost and time associated with producing the reagents needed for a given approach. Probe sets for FISH techniques, including fluorescence‐conjugated oligonucleotides and, in some approaches, primary encoding probes, may be major cost drivers for large screens. While small probe sets for ISS and simple linear FISH techniques are relatively inexpensive and straightforward to design, source, and inventory, investments in larger probe sets, such as those needed for transcriptome‐wide or large subsets like the L1000 transcript set (Subramanian *et al*, 2017), may be justified by their applicability to more types of studies, offsetting far greater upfront cost and effort through amortization across multiple screening projects.

### New biological model systems

While our group's primary focus has been the application of optical pooled screens in cancer cell lines, these systems adequately model only a subset of the biology we and others explore. For a given biological question, cancer cell lines may lack relevant genetic background or poorly represent a cell type of interest or epigenetic state. Further, most 2D cell culture systems are ill suited to the exploration of cell function in tissues and developmental trajectories. Fortunately, ISS approaches have been extended to primary human cells, organoids, and tissue sections. The extension of optical pooled screening approaches to these systems will expand the range of biological questions accessible to systematic screening approaches.

Extending optical pooled screens to primary cells will require application‐specific optimizations. In particular, some cell types may require immortalization to allow expansion or extended *in vitro* culture times to enable screening. Cell engineering for perturbations (e.g., Cas9 activity for gene knockout), perturbation barcode expression and *in situ* detection, phenotypic assay development, and computational tasks, such as cell segmentation, may pose specific challenges in primary cell systems, in particular heterogeneous performance across the population, demanding greater cellular throughput to maintain statistical power.

Organoid and *in vivo* screens face their own challenges for *in situ* detection of barcodes. Imaging in organoids requires overcoming poor reagent penetration into 3D multicellular structures, increased fluorescence background, and the requirement for increased z‐plane optical sectioning for phenotyping and possibly genotyping. Conversely, phenotyping is well‐established in thin tissue sections, and ISS has been demonstrated but perturbation barcode recovery from tissue sections at large scales still faces similar challenges including tissue autofluorescence and poor barcode detection efficiencies (Ke *et al*, 2013; Wang *et al*, 2018c). Some of these issues may be addressed through the implementation of tissue clearing, tissue expansion, and other genotyping protocol optimizations (Chung *et al*, 2013; Chen *et al*, 2015; Alon *et al*, 2021). Despite these challenges, Dhainaut *et al* (2022) have conducted targeted *in vivo* pooled screens with protein barcodes, taking advantage of the compatibility of IF with tissue sections to recover genotypes through iterative IF and MIBI‐TOF.

### Analysis methods

Much work is needed to capture the unique opportunities offered by profiling screens generally, and image‐based profiling screens in particular. Single‐cell resolution, and, for microscopy‐based profiling, spatial resolution, provide the opportunity to account for confounding variables such as cell state, local spatial context, and perturbation status (Dixit *et al*, 2016; Duan *et al*, 2019; Papalexi *et al*, 2021; Wang, 2021). For example, an analysis approach termed mixscape was applied to an scRNA‐seq pooled screen to reduce the contribution of confounding variables like cell cycle stage by comparing each perturbed cell to its nearest neighbor unperturbed cells (Papalexi *et al*, 2021). With spatial resolution, analogous corrections could be performed for covariates such as local cell density, well position, and more. Additionally, in contrast to enrichment screens where a single phenotype of interest is defined prior to screening, profiling screens provide the opportunity to explore genotype–phenotype associations at the analysis stage. With high‐dimensional single‐cell resolved phenotypic measurements, perturbations can be tested for association with any number of individual features or feature combinations. In addition, more rigorous statistical testing frameworks that account for (and test for) single‐cell variability in high‐dimensional spaces are needed. Finally, given the increasing wave of interest in profiling screens, their wide applicability, and the possibility of much higher throughput implementations, future analysis frameworks need robust and efficient scaling to billions of cells.

### Technology accessibility

Finally, improving technology accessibility is critical to establish optical pooled screening and to realize the wide impact of deploying this technology across a range of samples and disease areas. We parameterize “accessibility” for data generation and analysis by ease of use, consumables costs, and requirements for specialized equipment, reagents, and expertise. We see opportunities to improve the accessibility of optical pooled screens by improving screening efficiency and throughput to reduce required screening time and scale, reducing the cost of reagents for sequencing and phenotyping, simplifying and standardizing lower‐cost instrument configurations, implementing automated imaging and chemistry, and the availability of approachable, automated, and high‐performing yet flexible image analysis solutions and downstream analysis tools.

The first challenge new users encounter in performing an optical pooled screen is often assay validation in the desired biological model in combination with cell engineering and protocol requirements for genomic perturbation and *in situ* genotyping. These steps specifically include verifying the capacity to deliver perturbation reagents and effect perturbations, measure phenotypes, and recover perturbation barcodes at quantitatively adequate performance levels. Reagent databases, especially those validating antibody performance in IF applications, may be valuable resources in developing a given phenotyping panel. While each screen will still require application‐specific optimizations, continuing validation of perturbation and detection reagents by the research community across a wide range of biological systems and ongoing demonstrations of compatibility with *in situ* genotyping will gradually facilitate the development of new optical pooled screens.

Optical pooled screens provide a relatively affordable and approachable workflow for multidimensional profiling screening at high cellular throughput. However, lowering reagent costs and improving ease of use for ISS and phenotyping approaches would further improve accessibility and expand achievable scales. Currently, sequencing by ligation and SBS approaches have been demonstrated for ISS genotyping. Sequencing by ligation protocols use commercially available dye‐conjugated oligonucleotides and enzymes. We and others have used commercially available Illumina SBS reagent kits designed for four‐color readout on instruments such as the Illumina MiSeq (Payne, 2017; Chen *et al*, 2018; Feldman *et al*, 2019). Today, alternative SBS chemistries with fluorescent readouts are becoming commercially available from companies including Singular Genomics, Element Biosciences, and MGI. Concurrently, multiplexed *in situ* phenotyping approaches are likely to become more available and affordable as these approaches become more popular and the expanding market spurs commercialization. Several currently marked options span the range of phenotyping approaches discussed and include HCR probes for amplified linear FISH (Molecular Biotechnologies), MERFISH probes for exponential FISH (Vizgen), and oligo‐conjugated antibodies for Immuno‐SABER or CODEX iterative IF (several vendors).

Access to instrumentation, including high‐throughput screening microscopes and automated fluidics solutions, is a significant hurdle to implementing microscopy‐based screening. We estimate that an optical pooled screening microscopy setup similar to those used in our lab, including wide‐field fluorescence microscope, illuminator, camera, and appropriate filter sets, has a cost range of $100,000–$200,000 (USD), possibly higher if optical sectioning with confocal or light sheet imaging is required for assays. Like other major capital equipment for biological research, justifying this cost in most organizations depends on a plan for efficient utilization of the equipment by large projects or sharing instrumentation across many small projects. In addition, highly configured research microscopes are complex custom instruments, and while the hardware and software can be supported by commercial vendors, acquisition timelines, integration, setup, maintenance, robustness, and support are inferior in comparison with single‐configuration mass‐produced scientific instrumentation like dedicated sequencing instruments. “High content” screening microscopes are a more highly integrated option, but capital cost can be roughly an order of magnitude higher and may restrict sample type compatibility or desirable modification or integration with other automation systems. Finally, a new crop of integrated fluorescence imaging instruments with fluidics automation are nearing the market (Nanostring GeoMx, 10X Genomics Xenium) and may be adaptable for optical pooled screens.

Economic analysis of instrument productivity highlights the importance of imaging time in determining overall cost and throughput. Protocol automation, through integrated microscopy and fluidics, is an important driver for efficient utilization of equipment and labor, but poses additional engineering requirements. Custom built fluidics solutions have been developed to automate chemistry on microscopy samples (Gut *et al*, 2018; Almada *et al*, 2019; Eng *et al*, 2019; Xia *et al*, 2019b). Hurdles to implementing automated ISS and phenotyping include meeting the high degree of chemistry and imaging reliability needed for high‐throughput applications and managing workflows requiring staggered processing of multiple samples. Notably, there is basic compatibility of pooled profiling screening with the capability of commercial high‐throughput sequencing instrumentation. Many sequencers developed for SBS with fluorescent readout have advanced and high‐throughput imaging and reagent delivery capabilities, offering another path for repurposing mass‐produced commercial instrumentation for optical pooled screening. Challenges with adapting such instruments for optical pooled profiling screens include lack of support for user defined workflows and proprietary sample formats that may not be suitable for cell‐ or tissue‐based experiments. Several groups have repurposed retired four‐color sequencing instruments for optical screening (Uemura *et al*, 2010; Nutiu *et al*, 2011). For example, Pandit *et al* (2022) recently demonstrated automated chemistry and imaging for 4i multiplexed IF on an essentially unmodified Illumina HiSeq 2500 instrument. Largely replaced by higher throughput sequencers, retired HiSeq systems are currently available on the used market at substantially lower cost than new research microscopes.

## Disease applications

While we envision that optical pooled screening will help answer a diversity of biological research questions, we highlight here several areas where optical screens are particularly exciting for the study of human disease. Cell‐based imaging assays are already critical tools for the evaluation of gene function and compound mechanism of action (MOA) in modern drug discovery programs, leveraging a wide variety of labeling chemistries and imaging techniques to measure diverse molecular and cellular properties—particularly intermediate disease phenotypes visible in cells and reliably associated with disease etiology in humans. The relevance of *in vitro* imaging assays have been bolstered in recent years by advances in physiologically relevant model systems and advanced image analysis. In this section, we highlight the potential for optical pooled screens to elucidate mechanisms of disease pathogenesis, focusing on illustrative examples in cancer, infectious disease, and neurological disorders.

Ideal cancer therapies selectively kill tumor cells while minimizing adverse effects on the patient. Unfortunately, high‐throughput drug screens relying on cytotoxicity readouts have generally returned the same “low‐hanging fruit”—previously known MOAs that are highly druggable, such as microtubule dynamics and DNA modification (Moffat *et al*, 2014), slowing the discovery of selective therapies that address new tumor‐specific MOAs. Recently, NGS‐based methods for CRISPR screening and drug susceptibility profiling have made it possible to analyze cancer vulnerabilities at unprecedented throughput while gene expression profiling has enabled unbiased investigations of drug mechanisms (Barretina *et al*, 2012; Stransky *et al*, 2015; Iorio *et al*, 2016; Yu *et al*, 2016; Subramanian *et al*, 2017; Tsherniak *et al*, 2017; Ghandi *et al*, 2019; Corsello *et al*, 2020; McFarland *et al*, 2020; Srivatsan *et al*, 2020). Image‐based profiling is a complementary approach that has been used to map drug‐gene interactions and mechanisms of cytotoxicity (Breinig *et al*, 2015; Way *et al*, 2021). Targeted assays may also be used to examine cancer‐relevant phenotypes such as signaling dynamics, kinase activity, DNA damage, glucose consumption, and epithelial‐to‐mesenchymal transition (Kim *et al*, 2011; Purvis *et al*, 2012; Regot *et al*, 2014; Lotz‐Jenne *et al*, 2016; Ghezzi *et al*, 2019; Goglia *et al*, 2020). Pooled screening approaches facilitate the deployment of these assays across large libraries of genetic perturbations and multiple cellular models. We have demonstrated *in situ* genotyping of sgRNAs in tens of cancer cell lines, with effective genotyping typically possible using a single, standard protocol (Feldman *et al*, 2022).

There is significant interest in using optical pooled screens to study the effects of genetic perturbations in organoid models and tissue sections. Recently, Dhainaut *et al* (2022) used protein barcodes to analyze the effects of gene knockouts on phenotypes measured *in vivo*, including tumor growth, immune composition, and gene expression.

Optical methods have also served a crucial role in the analysis of host‐pathogen interactions, enabling precise studies of pathogen life cycle, the suite of host antiviral responses, and classification of diverse infection outcomes. For example, high content assays have been developed to study individual steps during viral infection, including viral entry, unpackaging and gene expression (Karlas *et al*, 2010; Banerjee *et al*, 2013). This contrasts with many other infection screening approaches that provide a unidimensional readout of viral binding, entry, or cytopathic effect alone. Amidst the ongoing SARS‐CoV‐2 pandemic, imaging approaches have been used to identify compounds with antiviral properties and to model the effects of cytokine storm (preprint: Cuccarese *et al*, 2020; Zhu *et al*, 2020).

Meanwhile, antibiotic resistance has stimulated interest in therapeutics that target host pathways instead of bacteria directly. An RNAi screen of the human kinome uncovered several kinases that inhibit the growth of *Salmonella* and follow‐up studies linked phagosome maturation in host cells with pathogen survival (Kuijl *et al*, 2007). Imaging has also been used to dissect microbe genetics. For example, an arrayed Mtb transposon screen identified mutants with impaired capacity to survive inside host macrophages (Barczak *et al*, 2017). Imaging is also a powerful tool to probe cell non‐autonomous phenotypes, such as cell–cell interactions and tissue‐level organization of immune cells, which play important roles in pathogen responses (Pasqual *et al*, 2018; Gola *et al*, 2021).

Neurological disorders are characterized by a number of phenotypes that are ideally suited to image‐based readout, such as neuron morphology, protein and RNA aggregation, synapse formation, and electrical function, phenotypes often most relevant in the context of spatial and functional relationships among cells (Sepp *et al*, 2008; Linhoff *et al*, 2009; Jain & Vale, 2017; Kiskinis *et al*, 2018; Tian *et al*, 2019; Duan *et al*, 2021). Many cells in neural tissues have extended morphologies, including long processes where many such critical functions and interactions are localized. The requirements for cell dissociation and handling of flow cytometry and single‐cell NGS methods are often incompatible with cells from neural tissues, limiting researchers to discern what they can from cell nuclei stripped of most functional cellular components. Optical profiling screens offer opportunities to study intact interacting cells localized in or obtained from neural tissues. Pooling offers a straightforward strategy to scale up assays for neuronal phenotypes across large genomic libraries, yet presents a number of challenges.

Cell segmentation is particularly difficult in cell types with complex morphologies and long processes like neurons, making it difficult to associate cell bodies and nuclei with more distal parts of the same cell where many activities of interest manifest. For optical pooled screens, this impacts both phenotypic imaging and the association of phenotypes at the cell periphery with perturbation identities. Neuron barcoding and machine learning approaches offer two potential classes of strategies for improved segmentation, but have yet to be fully developed or integrated with optical profiling screens (Ronneberger *et al*, 2015; Wroblewska *et al*, 2018; Chen *et al*, 2019; Moen *et al*, 2019). The establishment of readily engineerable disease‐relevant model systems, such as induced pluripotent stem cell‐derived neurons, as validated models for optical pooled screens is another priority for technology development in this area. For optical pooled screens, this requires the demonstration of efficient perturbation delivery and *in situ* detection in neural cell types. As optical screens move into organoids and solid tissues, the brain is an attractive system for whole‐mount imaging due its relative transparency compared with other tissue types.

## Conclusion

The development of programmable genetic perturbation technologies, microscopy‐based high‐dimensional phenotypic assays, and screening methodologies to associate perturbations to phenotypes have created exciting opportunities to study genotype–phenotype relationships with genetic screens. We anticipate that these technologies will mature and be integrated in approachable and accessible screening workflows at very large scales that enable the broader research community to routinely access high‐dimensional and single‐cell resolved readouts for genome and epigenome scale perturbation screens as well as new use cases yet to be imagined.

## Author contributions

**Russell T Walton:** Writing – original draft; writing – review and editing. **Avtar Singh:** Writing – original draft; writing – review and editing. **Paul C Blainey:** Writing – original draft; writing – review and editing.

## Disclosure and competing interests statement

The Broad Institute and MIT have filed patent applications on work described in this manuscript and may seek to license the technology. PCB and AS are listed as inventors. PCB is a consultant to and/or holds equity in companies that develop or apply genomic or genome editing technologies: 10X Genomics, General Automation Lab Technologies, Celsius Therapeutics, Next Gen Diagnostics LLC, Cache DNA, Concerto Biosciences, and Stately Bio. PCB's laboratory receives funding from industry for related work from Merck and Calico Life Sciences.
